# Analysing monkeypox epidemic drivers: Policy simulation and multi-index modelling across 39 nations

**DOI:** 10.7189/jogh.14.04037

**Published:** 2024-02-09

**Authors:** Mengxuan Lin, Yingrong Xin, Jiaojiao Wang, Pengyuan Nie, Qunjiao Yan, Ligui Wang, Lei Wang

**Affiliations:** 1 Academy of Military Medical Sciences, Academy of Military Science of Chinese People’s Liberation Army, Beijing, China; 2 Chinese People’s Liberation Army Center for Disease Control and Prevention, Beijing, China; 3 State Key Laboratory of Multimodal Artificial Intelligence Systems, Institute of Automation Chinese Academy of Sciences, Beijing, China

## Abstract

**Background:**

This study aimed to analyse the drivers of the monkeypox (Mpox) epidemic and policy simulation to support health care policies against the Mpox epidemic.

**Methods:**

We established a three-round selection mechanism for 164 factors using Lasso and negative binomial regression to investigate the correlation between significant drivers and the cumulative confirmed cases of Mpox. Policy simulation for each driver was evaluated, and the varying effects of implementation at different times were examined.

**Results:**

HIV/AIDS prevalence and air transport passengers carried were significant determinants of the risk of the Mpox epidemic across various countries, with regression coefficients of 1.417 and 0.766, respectively. A decrease in HIV/AIDS prevalence by 10, 20, 30, and 40% corresponded to reductions in the number of Mpox cases by 6.28, 6.55, 6.87, and 7.26%, respectively. Similarly, 20, 40, 60, and 80% travel restrictions led to reductions in Mpox cases by 7.16, 15.63, 26.28%, and 41.46%, respectively. Controlling air transport passengers carried in the first month could postpone outbreak onset by 0.5–2.0 months.

**Conclusions:**

Mpox prevention and control policies should primarily focus on travel restrictions during high disease-risk periods and flight suspensions from high-risk nations in combination with regular HIV/AIDS prevention and treatment strategies.

Monkeypox (Mpox), a viral zoonotic disease characterised by chills, fever, swollen lymph nodes, and a distinctive rash, has gained global attention since its initial identification in 1958 during research on primates imported from Singapore. The virus, known as the monkeypox virus (MPXV), has led to sporadic human cases in various African countries since the 1970s [1–3].

The global Monkeypox (Mpox) pandemic, emerging on 7 May 7 2022, with the first reported case in the UK [4], swiftly evolved into a worldwide crisis, prompting the World Health Organization (WHO) to declare it a Public Health Emergency of International Concern (PHEIC) on 23 July 2022 [5,6]. By 3 May 2023, Mpox had resulted in 87 301 confirmed cases and 130 fatalities across 111 countries [7].

Despite the rising incidence of Mpox and the identification of distinct MPXV clades in Central and West Africa, the transmission dynamics of the disease and factors influencing its prevalence remain elusive [8–13]. Previous studies have demonstrated a significant increase in human-to-human transmission, especially in regions outside Africa [14,15]. The recent Mpox pandemic underscored the urgency of understanding and controlling the disease, especially in the context of the COVID-19 pandemic [16].

This study aims to bridge critical gaps in our understanding of Mpox, focusing on factors influencing its prevalence and the effectiveness of control measures. By investigating socioeconomic, environmental, and meteorological variables across 39 countries, we aimed to identify key indicators impacting Mpox outcomes.

While global control measures during the pandemic were successful, variations in disease duration and new cases following domestic outbreaks suggest the influence of additional factors. This study adopts a comprehensive approach, considering indicators such as per capita gross domestic product (GDP), income, number of hospital beds, and the Air Quality Index, aiming to provide valuable insights for public health decision-making.

## METHODS

### Data source

This study used data on Mpox cases, prevention and control policies, and socioeconomic, environmental, and meteorological factors (Tables S3–S5 in the **Online Supplementary Document**) from 39 countries where the cumulative total number of confirmed cases had surpassed 50 [7] by 19 February 2023. A total of 164 factors that may potentially influence Mpox transmission were analysed. The data for Mpox prevention and control policies were consolidated from information published by national public health departments and news portals. Data on Mpox cases were retrieved from the WHO website [7]. The socioeconomic, environmental, and meteorological factors were identified from ‘Our World In Data’ [17]. The factors were categorised into five main areas: health (39), disease (20), livelihood (78), economy (14), and environment (13). These factors have been either proven or hypothesised to affect the prevalence of Mpox and other infectious diseases and include air pollution [18], medical resources [19], income level [20], vaccination [21], travel [22], and the incidence of other diseases related to Mpox [23,24].

Due to the significant differences in the absolute values of socioeconomic, environmental, and meteorological factors among different countries, we standardised these factors into averages or rates to ensure the scientific validity and accuracy of our model.

### Model overview

Due to the substantial collinearity among the socioeconomic, environmental, and meteorological factors (Figures S2–S6 in the **Online Supplementary Document**), this study implemented three rounds of variable selection. First, L1 regularisation was applied separately to each data category. Next, regularisation was applied to all significant output factors without any categorisation. Finally, negative binomial regression analysis was applied to factors exhibiting statistical significance, with Mpox cases as the dependent variable. These three rounds of variable selection eliminated most of the 164 factors, thus enhancing the precision, scientific validity, and interpretability of the model's analytical outcomes.

The first and second rounds of variable selection were guided by Lasso regression, specifically utilising Lasso regression with categorical variables as an L1 regularisation mathematical model. Regularisation is a general term for a class of methods in machine learning and introduces additional information to the original loss function to prevent overfitting and enhance the generalisation performance of the model. It includes both L1 regularisation (Lasso regression) and L2 regularisation (ridge regression). The norms produced from these methods serve as the penalty terms for the loss function [25]. In contrast to L2 regularisation, which is suitable for addressing overfitting issues, L1 regularisation is particularly proficient at selecting multi-dimensional variables with a degree of collinearity. The loss function for Lasso regression with categorical variables can be defined as follows:



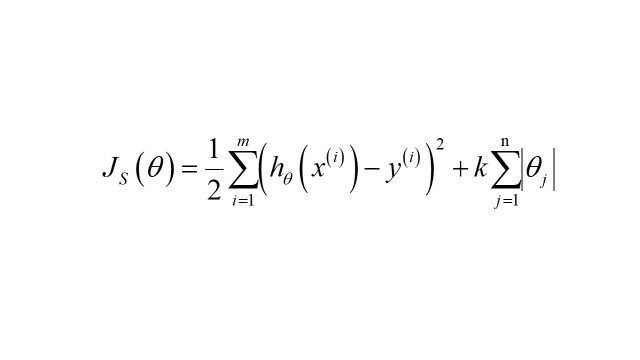



where *S* denotes the category of socioeconomic, environmental, and meteorological factors.The value of *k* (0<*k*<1) was determined to strike a balance between variance and bias; the increase in *k* was accompanied by a decrease in model variance and an increase in bias. An excessive *k* value could lead to overfitting. A learning rate of 0.01 was set in this study, and the *k* value was gradually increased from zero. Cross-validation was performed to test the error on the test set. This process was iterated until the test set error was minimised.

Generalised linear models are typically used for infectious disease analysis and prediction [26]. The incidence and prevalence of Mpox often exhibit geographical spatial aggregation, failing to meet the strict Poisson distribution and independence principles of test methods. The data showed over-dispersion, with the variance significantly exceeding the mean (Table S6 in the **Online Supplementary Document**). This over-dispersion can underestimate the standard errors when estimating model parameters using Poisson regression. In our study, this underestimation resulted in false positives for socioeconomic, environmental, and meteorological factors. Additionally, the prerequisites for using negative binomial regression include an absence of multicollinearity among the independent variables. Given these circumstances, negative binomial regression was deemed a more suitable tool and selected as the model for driver analysis in the third round of variable selection. The equation of negative binomial regression is as follows [27]:


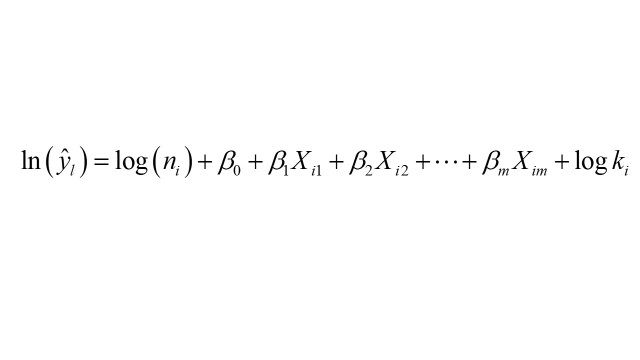
,

where log(*n_i_*) is the offset, β_0_ is the intercept, *β_m_* is the regression coefficient of *X_im_*, and *k_i_* is the degree of dispersion of *X_i_*.

### Model validation

The dimensionality reduction accuracy of the data through Lasso regression could be intuitively observed by the trend of the standardised regression coefficients of variables approaching 0 as the value of *k* increased. Following two rounds of variable screening, a third round of driver screening for 39 countries was conducted using negative binomial regression, which also served to analyse the epidemic risk of Mpox. The model calculation results were compared with the actual case data to calculate the error. The model validation methods included the likelihood ratio test and the Akaike and Bayesian information criteria. The validation analysis revealed that the model performance of Lasso regression and negative binomial regression was within an acceptable range. Further policy simulation analysis was conducted based on this result. The analysis for this study is based on Python 3.9 and IBM SPSS Statistics 26 (IBM, USA).

## RESULTS

**Figure 1:**
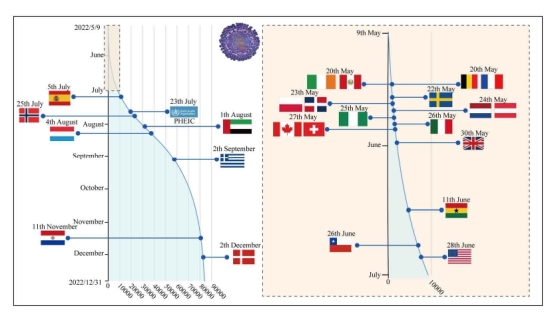
Timeline of global cumulative cases and first implementation of monkeypox (Mpox) health policies in various countries. The y-axis represents time, and the x-axis represents the number of Mpox cases. The pale yellow box on the right is a close-up view of the left side from May to July 2022. The indicated times represent instances when countries initially implemented control guidelines, isolation measures, or other policies relevant to Mpox. The data were obtained from government and public health department websites and news media reports (Table S3 in the **Online Supplementary Document**).

The first case of the present Mpox pandemic was reported in the UK on 7 May 2022, followed by a sharp initial increase in cases, with more than 80 000 reported cases in 2022. Several countries in Europe and North America issued prevention guidelines and isolation measures within approximately ten days of their first registered case. Moreover, almost all announcements were made within the first month of the global outbreak of Mpox (May 2022). Belgium was the first country to mandate isolation for patients with Mpox [28]. The preventive measures these countries put in place proved ineffective, as indicated by the sharp rise in cases, which peaked at more than 6500 new cases per week three months later (**Figure 1**). The Mpox outbreak began to subside in October 2022 with a slow yet steady decline to below 200 new cases by February 2023 (Table S4 in the **Online Supplementary Document**).

**Figure 2:**
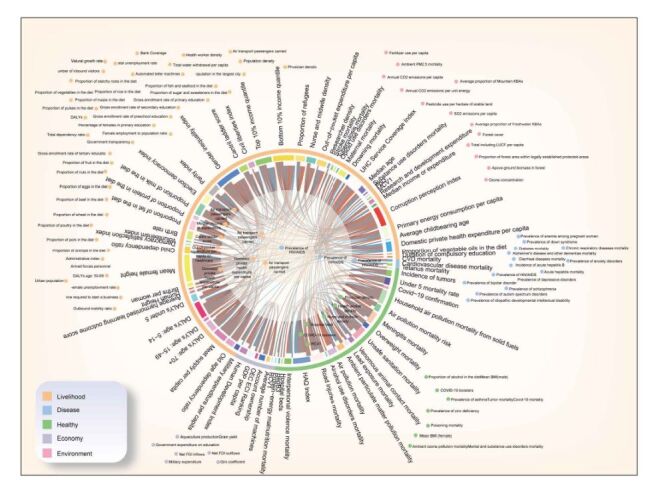
A schematic diagram of multicollinearity among factors and the three rounds of variable selection. The five different colours on the outermost circle represent different categories of factors. The discontinuous colour bands inside indicate a high correlation between individual factors and other factors of the same category. The length of the colour band is proportional to the quantity of highly correlated factors. Lines connect highly correlated factors, with grey and brown lines indicating positive and negative correlations, respectively. Scattered points outside the circle are factors that do not correlate highly with other factors. The factors on the lines, white ring, and orange ring are significant after the first, second, and third rounds of variable selection, respectively.

Within the 164 factors analysed, a high correlation was observed among factors within the same category, leading to as many as 220 groupings of highly correlated factors (with the absolute value of the Spearman correlation coefficient |*ρ_S_*| exceeding 0.8) (**Figure 2**). Mean female height, the gender inequality index, the lowest 10% income quantile, and the corruption perception index correlated highly with over 15 other factors, showing extreme collinearity. An initial round of variable selection was performed to mitigate this, with each category of factors selected via Lasso regression. Out of the 164 factors, 12 were significant (Table S5 in the **Online Supplementary Document**). This allowed for considerable data dimension reduction, effectively addressing the collinearity issue. This outcome was also determined by the results of the copula process and the variance inflation factor (VIF) test (Tables S2 and S7 in the **Online Supplementary Document**). Of the 12 factors considered significant in the first round of selection, 6, 3, and 1 factor(s) fell in the categories of health, livelihood, and disease, respectively. All factors pertaining to economic and environmental categories were subsequently excluded.

**Figure 3:**
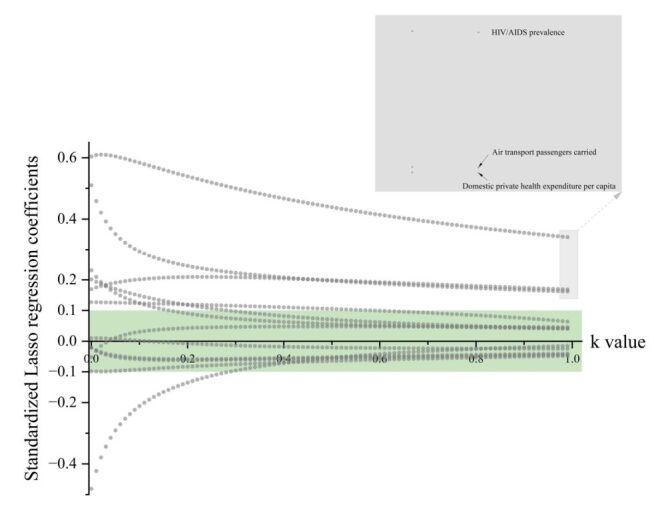
The second round of variable selection with Lasso regression. The y-axis represents the standardised regression coefficients of each factor, and the x-axis represents the value of *k*. As the value of k increases and approaches 1, the green band serves as the threshold region to determine whether the regression coefficient approaches 0. DRC – The Democratic Republic of the Congo

**Figure 4:**
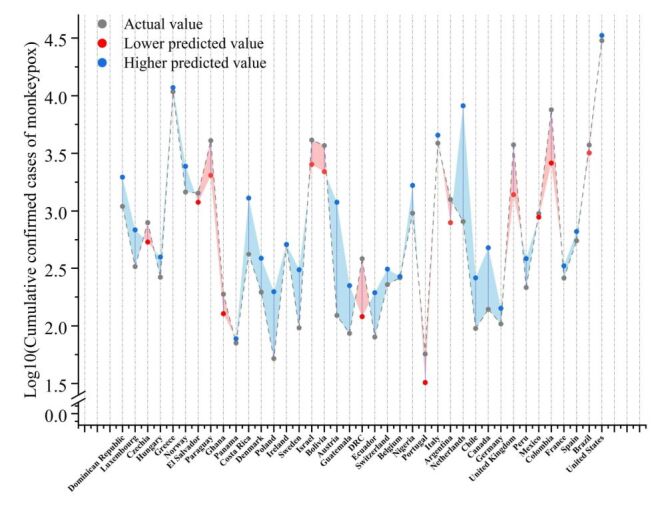
Validation of the negative binomial regression model. The y-axis represents the cumulative confirmed cases of monkeypox (Mpox) after logarithmic transformation, and the x-axis represents the countries. The red and blue areas are defined by the area between the connecting lines where the predicted values are lower and higher than the actual values, respectively.

During the second selection round, a Lasso regression was performed on the significant factors identified in the first round without any categorisation. Only three factors, namely, HIV/AIDS prevalence, air transport passenger carried, and domestic private health expenditure per capita, were retained, among which two were livelihood factors and one a disease factor (**Table 1**). The regression coefficients of the remaining nine factors approached 0 as the *k* value increased (**Figure 3**). Finally, only HIV/AIDS prevalence and air transport passengers carried effectively influenced the cumulative cases of Mpox in the third round of the negative binomial regression prediction model (**Table 2**). These two factors fell into the categories of disease and livelihood, respectively, both positively impacting the cumulative confirmed cases of Mpox, with regression coefficients of 1.417 and 0.766, respectively. In terms of model validation, the predictive model, established using the two-factor negative binomial regression, demonstrated satisfactory fit and statistical significance (**Figure 4**, **Table 3**), confirming the feasibility of the model.

**Table 1:** Summary of Lasso regression performance in the second round of variable selection

Factor	Unstandardised coefficient		Standardised coefficient	*T-*value	*P*-value
	***B***	**SE**	***Beta***		
**Constant**	-5013.963	6633.891		-0.756	0.456
**Livelihood**					
Physician density	-54.915	389.124	-0.021	-0.141	0.889
Nurse and midwife density	-51.184	206.820	-0.052	-0.247	0.806
Health worker density	215.277	129.014	0.201	1.669	0.107
Median income or expenditure	55.374	46.801	0.232	1.183	0.247
Out-of-pocket expenditure per capita on health care	-6.032	2.965	-0.481	-2.034	0.052
Domestic private health expenditure per capita	2.230	1.020	0.510	2.186	<0.05
Air transport passenger carried	980.710	451.052	0.210	2.174	<0.05
**Health**					
Hospital beds	24.748	263.374	0.010	0.094	0.926
COVID-19 boosters	-4.141	24.326	-0.021	-0.170	0.866
RCV1	-56.383	61.060	-0.098	-0.923	0.364
Disease					
HIV/AIDS prevalence	2866.474	589.469	0.604	4.863	<0.01

COVID-19 – coronavirus disease 2019, RCV1 – rubella-containing vaccines I, SE – standard error

**Table 2:** Summary of negative binomial regression performance in the third round of variable selection*

Factor	Regression coefficients	Standard error	*Z*-value	*P*-value	OR (95% CI)
**Livelihood**					
Domestic private health expenditure per capita	-0.001	<0.001	-0.097	0.922	1.000 (1.000–1.000)
Air transport passenger carried	0.766	0.213	3.592	<0.01	2.151 (1.416–3.268)
**Disease**					
HIV/AIDS prevalence	1.417	0.204	6.958	<0.01	4.123 (2.766–6.144)
Intercept	-0.658	1.338	-0.492	0.623	0.518 (0.038–7.136)

CI – confidence interval, OR – odds ratio

*McFadden R^2^ = 0.125.

**Table 3:** Likelihood ratio test of negative binomial regression model

Model	-2 log-likelihood	χ^2^ value	df	*P*-value	AIC value	BIC value
Intercept only	677.696					
Negative binomial regression model	593.200	84.497	3	<0.01	601.200	-106.866

AIC – Akaike information criterion, BIC – Bayesian information criterion, df – degrees of freedom

**Figure 5:**
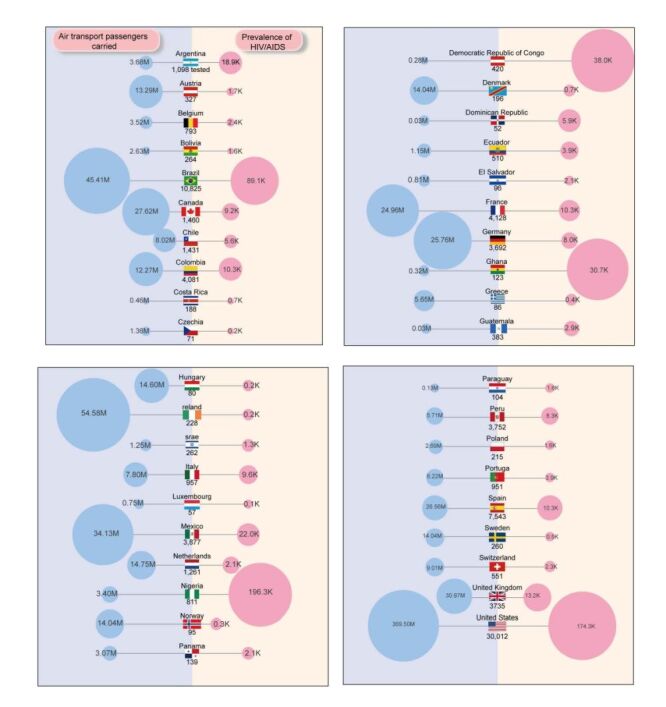
Values of the two factors significantly affecting the prevalence and the cumulative confirmed cases of monkeypox (Mpox) in 39 countries. The diameter of the circle is proportional to the numerical value. The number of cumulative confirmed cases of Mpox in the country is indicated below the corresponding flag.

**Figure 6:**
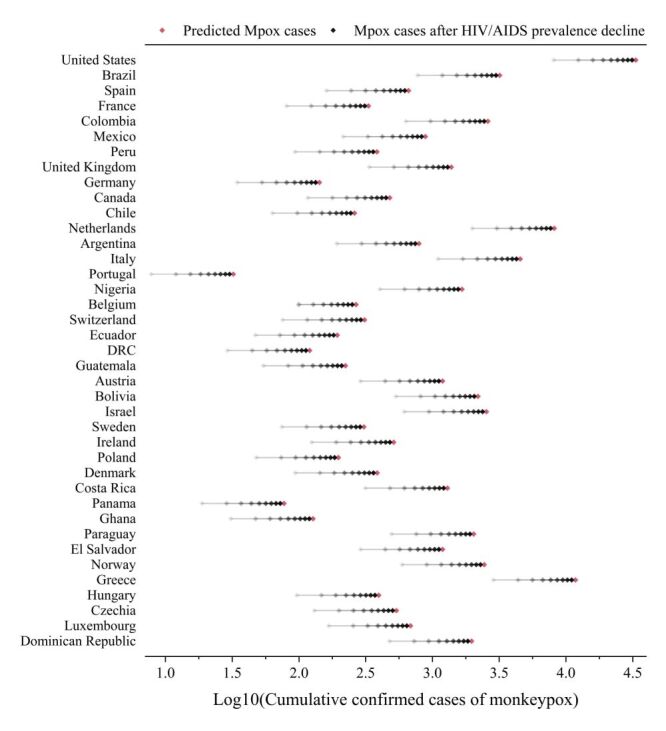
Mpox cases when HIV/AIDS prevalence is at varyingly low levels. The y-axis represents the countries, and the x-axis represents the cumulative confirmed cases of Mpox after logarithmic transformation. The red dots are the output by the predictive model of the cumulative confirmed cases of Mpox without any intervention measures, and the black dots are the cumulative confirmed cases of Mpox when HIV/AIDS prevalence is at varying low levels. The lighter the colour, the lower the HIV/AIDS prevalence, decreasing in 10% increments. Mpox – Monkeypox

Significant differences were observed in the HIV/AIDS prevalence and air transport passenger carried across 39 countries (**Figure 5**). This led to differences in the implementation challenges and efficacy of measures targeting Mpox among these countries. HIV/AIDS prevalence serves as a long-term indicator; short-term implementation of Mpox outbreak control policies would not result in a significant reduction. In this study, we simulated the number of Mpox cases in 39 countries prior to the Mpox outbreak, considering different relatively low levels of HIV/AIDS prevalence (**Figure 6**). With each 10% reduction in HIV/AIDS prevalence, there was a decrease in the cumulative confirmed cases of Mpox, ranging from 6.28% to 12.90%. A nonlinear positive correlation was observed between the two variables, with a greater reduction in Mpox cases occurring at lower levels of HIV/AIDS prevalence, indicative of a magnifying effect. Considering a realistically achievable reduction range, which includes a decrease in HIV/AIDS prevalence by 10, 20%, 30, and 40%, the number of Mpox cases decreased by 6.28, 6.55, 6.87, and 7.26%, respectively.

**Figure 7:**
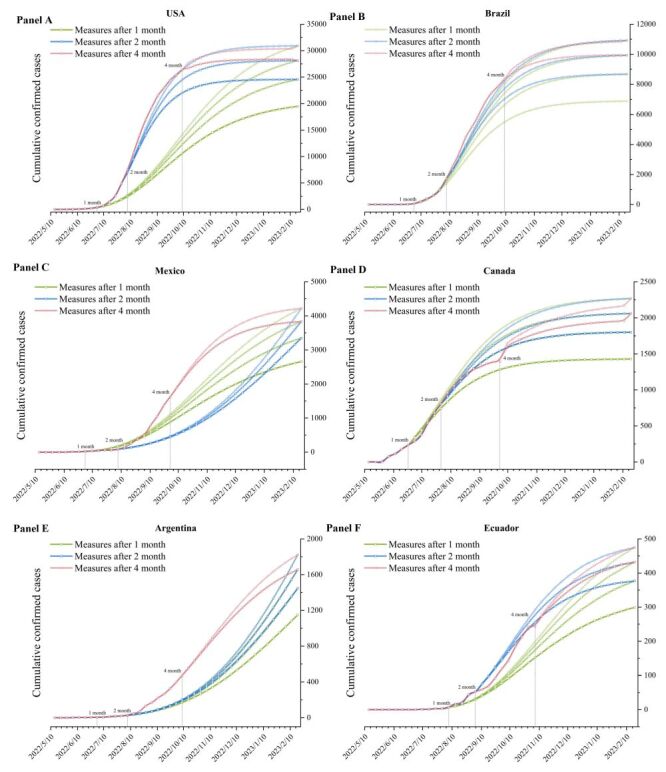
The prevalence of monkeypox (Mpox) when travel restrictions were implemented at different times in specific countries. **Panel A.** USA. **Panel B.** Brazil. **Panel C.** Mexico. **Panel D.** Canada. **Panel E.** Argentina. **Panel F.** Ecuador. The y-axis represents the cumulative confirmed cases of Mpox, and the x-axis represents the dates. Green, blue, and red lines are the fitted curves of cases (the fitting function is 
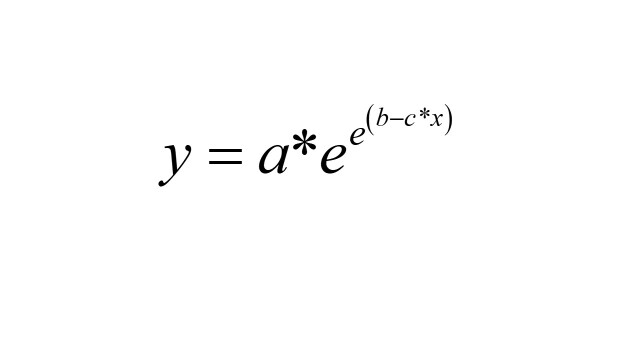
) after the implementation of travel restrictions one, two, and four months, respectively, after the report of Mpox cases, with a darker colour indicating stricter travel restrictions. The restriction levels were implemented for different durations: one month with levels at 20, 40, 60, and 80%; two months with levels at 20, 40, and 60%; four months with levels at 20 and 40%. Before the travel restrictions were enforced, air transport passengers carried remained at normal levels.

Travel restrictions emerged as the most prevalent measures nations took in response to the Mpox outbreak and can be implemented at any stage. We selected specific countries where the number of Mpox cases and the two drivers (HIV/AIDS prevalence and air transport passengers carried) were different to simulate the impact of enforcing travel restrictions at different times (**Figure 7**, panels A–F). It was clear that the timing of introducing travel restrictions could affect the eventual cumulative confirmed cases and the trajectory of Mpox incidence to different extents. When travel restrictions were implemented at levels of 20, 40, 60, and 80%, there was a decrease in the number of Mpox cases by 7.16, 15.63, 26.28, and 41.46%, respectively, suggesting a nonlinear positive correlation with the magnifying effect, similar to that observed with the HIV/AIDS prevalence factor. Moreover, in most countries, the earlier implementation of travel restrictions was correlated with a delay in the peak of the disease. Compared with initiating travel restrictions in four months, implementing these measures within 1 month could defer the peak of the disease by approximately 0.5–2.0 months.

## DISCUSSION

The findings of this study offer significant insights into the complex interplay of socioeconomic, environmental, and meteorological factors contributing to the Mpox epidemic. By incorporating 164 variables, our analysis provides a robust framework for understanding the drivers of Mpox and offers a novel approach to policy simulations that could be adapted for other emerging infectious diseases.

Countries impacted by Mpox typically launched their initial Mpox prevention and control guidelines, isolation measures, or related policies within one to two months of the first reported cases, which is reasonably prompt and proactive. Nevertheless, a substantial surge in Mpox cases occurs thereafter, with the disease spreading to other continents. Aside from the Mpox epidemic in May and June, which occurred during a period of rapid growth in infectious disease dynamics, another critical reason for this growth was that the primary policies adopted by most countries were centred around vaccination and community isolation [29]. These measures were unable to significantly affect the drivers contributing to the prevalence of Mpox.

After three rounds of variable selection and predictive modelling, we found that both the prevalence of HIV/AIDS and the air transport passengers carried significantly impacted the Mpox prevalence of a country. Therefore, implementing Mpox policies addressing these factors can achieve a more effective result. A principal strategy for managing the number of air transport passengers is to impose travel restrictions during high-risk periods and suspend incoming flights from high-risk countries. This strategy was applied to the prevention and control of most person-to-person transmitted infectious diseases and was utilised during the COVID-19 pandemic by many countries over a prolonged period, considerably reducing the number of imported cases [30]. However, given the lower pathogenicity and transmissibility of Mpox than those of COVID-19 and the fact that excessive population control measures caused great economic damage during the COVID-19 pandemic, these measures were not applied during the Mpox pandemic. Moreover, the timing of implementation of these measures can impact the trend of Mpox prevalence. Specifically, the earlier the travel restrictions are implemented, the more the surge in Mpox cases is delayed. A plausible explanation for this is that airport controls reduced imported cases and population movement. By preventing individuals infected overseas from entering domestic cities, these controls can effectively inhibit domestic transmission and delay the increase in local cases. Therefore, it is recommended that countries should promptly impose flight restrictions in the early stages of the domestic Mpox outbreak and implement airport lockdowns if necessary to minimise population movement.

There is a high possibility of sexual transmission of Mpox, and many cases involving Men who have sex with men (MSM) have been reported in various countries [7,31–35]. The prevalence of Mpox is closely related to the prevalence of HIV/AIDS, a finding confirmed by this study. Reducing HIV/AIDS prevalence requires a long-term approach based on prevention and treatment of the disease and cannot significantly change within a short timeframe following the prevalence of Mpox. As such, rapid suppression of Mpox prevalence via HIV/AIDS control is unrealistic.

We identified a moderate to high positive correlation between HIV/AIDS prevalence and numerous epidemic factors (Figure S3 in the **Online Supplementary Document**). Therefore, sustained control and a steady reduction of HIV/AIDS prevalence can aid in suppressing the high incidence of local epidemics and effectively reduce the intensity of future epidemic outbreaks. Prevention and control policies should focus on MSM, such as promoting fixed sexual partners, condom use, circumcision, and regular HIV testing.

This study is the first to utilise 164 socioeconomic, environmental, and meteorological factors to analyse the drivers of the Mpox epidemic and simulate policy evaluations of Mpox prevalence across 39 countries. Existing studies on Mpox drivers, limited by model scope and input variables, typically analyse between five and 20 drivers [29,36,37], with no variable selection conducted. This study proposed an innovative three-round variable selection strategy that effectively eradicates variable multicollinearity while maintaining substantial data volume to provide decision support for governmental bodies and public health departments and promote further research that applies this approach. This is because such driver analysis and policy simulation evaluations apply to most emerging infectious diseases.

The strong correlation between HIV/AIDS prevalence and Mpox incidence underscores a critical intersection of public health concerns. Policies aimed at mitigating HIV/AIDS could, therefore, have a dual benefit in reducing the susceptibility to Mpox, particularly among high-risk groups such as MSM. The potential application of these findings extends beyond immediate epidemic control and could lead to integrated health care strategies that address multiple infectious diseases concurrently.

Moreover, the study’s implications for air travel policies suggest that a revision of international health regulations may be warranted. Considering the balance between the economic impact of travel restrictions and their effectiveness in controlling disease spread, this study provides empirical support for the strategic timing of such measures. This could lead to the development of dynamic policy models that adapt to the real-time assessment of epidemiological risks.

While the three-round variable selection strategy mitigates the issue of multicollinearity and preserves data richness, the exclusion of variables through this process may inadvertently overlook factors with smaller but still significant effects on Mpox prevalence. Additionally, the study’s reliance on numerical data and mathematical modelling may not fully account for qualitative factors, such as cultural practices or the quality of health care systems, which could have a nonlinear impact on the spread of infectious diseases.

The study does not address the synergistic effects of multiple policies when implemented in concert, which is a common approach in public health interventions. The interaction between different policy measures may result in either compounding or diminishing returns, which is not captured in our current model.

Furthermore, the aggregation of data across 39 countries may mask regional or local variations that are critical to understanding and responding to the epidemic. The heterogeneity among these countries suggests that a ‘one-size-fits-all’ approach may be less effective than tailored strategies that account for specific local contexts.

Future research should aim to develop composite policy models that can simulate the combined effects of various interventions [38–40], which may provide a more accurate and practical guide for policymakers. Additionally, the integration of qualitative data through mixed-methods research designs may yield a more nuanced understanding of the factors influencing Mpox prevalence [41].

The variation in driver values across countries suggests that future studies may benefit from country-specific analyses (**Figure 5**). This will allow for the design of locally optimised policies and potentially uncover region-specific drivers that are not apparent in a broader analysis [42,43].

In conclusion, while this study advances the understanding of Mpox drivers and policy implications, it is imperative that future research builds upon the limitations identified, incorporating a more granular approach and considering real-world complexities of health policy implementation [44–46].

## CONCLUSIONS

The prevalence of HIV/AIDS and the number of air transport passengers significantly impact the prevalence of Mpox. A simultaneous reduction in these two factors may decrease the cumulative cases of Mpox. A greater delay in Mpox peak incidence can be achieved when control measures are implemented early. Therefore, governments should develop Mpox policies that consider the acceptability of these measures, including travel restrictions during high-risk periods and flight bans from high-risk countries. These measures, combined with routine HIV/AIDS prevention and treatment strategies, can enable effective prevention and control of Mpox.

## Additional material


Online Supplementary Document
