# Supplementary material for: Analysing monkeypox epidemic drivers: Policy simulation and multi-index modelling across 39 nations
Source: J Glob Health. 2024 Feb 9;14:04037. doi: 10.7189/jogh.14.04037 (PMC10859682; doi:10.7189/jogh.14.04037)
Supplement: Online Supplementary Document [file jogh-14-04037-s001.pdf]

Supplementary Materials

Copula process in the second round of variable selection

When the data of each dimension are not independent of each other, modelling the joint distribution is challenging. Under these circumstances, the Copula function, which can be used to model the correlation of random variables with known marginal distributions, is a widely utilised tool.

The concept of the Copula function was introduced in 1959 by Sklar through a theorem according to which the joint distribution of N random variables can be expressed using their respective marginal distributions and a copula function. This separates the randomness of the variables and their coupling. The randomness of the variables is characterised by the marginal distribution, while the coupling is by the copula function [1-3]. In general, the correlation properties of a joint distribution are entirely governed by its copula function. The copula function equation can be expressed as follows:

$$C(u,v) = H(F^{-1}(u), G^{-1}(v)),$$

where $F(u)$ and $G(v)$ are continuous edge distributions, and $H(u,v)$ is the binary joint distribution function of $F(u)$ and $G(v)$.

This study employed the group of elliptic cointegration functions to model the 12 dimensions of the second-round selected socioeconomic, environmental, and meteorological factors. This group encompasses the Student t copula and the Gaussian copula, both of which exhibit symmetrical tail correlation and have similar central areas, with the principal distinction lying in the thickness of the tails. The Gaussian Copula,

based on the parameter matrix R, can be represented as follows:

$$C_R^{Gauss}(u) = \Phi_R\left(\Phi^{-1}(u_1), \dots, \Phi^{-1}(u_d)\right)$$

The formula for the Student t copula function is as follows:

$$C_{v,\boldsymbol{\Sigma}}^{t}(\mathbf{u}) = t_{v,\boldsymbol{\Sigma}}\left(t_v^{-1}(u_1), \dots, t_v^{-1}(u_d)\right)$$

All copula functions must also satisfy the following conditions:

1. The definition domain is $[0,1] \times [0,1]$, the value range is $[0,1]$ ($C\colon [0,1] \times [0,1] \to [0,1]$)

2. $C[u,0] = C(0,v) = 0, C(u,1) = C(1,u) = u, C(v,1) = C(1,v) = v$

3. $0 \leq \frac{\partial C(u,v)}{\partial u} \leq 1, 0 \leq \frac{\partial C(u,v)}{\partial v} \leq 1$, or $C(u_2,v_2) - C(u_2,v_1) - C(u_1,v_2) + C(u_1,v_1) \geq 0$ ($0 \leq u_1 \leq u_2 \leq 1$ and $0 \leq v_1 \leq v_2 \leq 1$)

After calculating the marginal distributions for the 12-dimensional data, the Gaussian-copula was determined as the function to reflect the coupling relationship of the socioeconomic environmental and meteorological factors according to Akaike Information Criterion (AIC) and Bayesian Information Criterion (BIC) (Supplementary Table S1). We performed a visual analysis of the joint distribution of the two groups of variables with the highest correlation coefficients (Supplementary Figure S1). The application of the Gaussian copula function to the socioeconomic, environmental, and meteorological factors revealed a completely different coupling relationship from that observed in the initial data (Supplementary Table S2).

Supplementary Tables

Supplementary Table S1. Selection based on a group of elliptic cointegration functions

Function name	AIC	BIC
Student t copula	-374.67	-338.08
Gaussian-copula	-354.20	-317.60

Supplementary Table S2. Pearson correlation coefficient matrix after the copula process

Factor	Physician density	Health worker density	Nurse and midwife density	Hospital beds	COVID-19 boosters	RCV1	Median income or expenditure	Cantril ladder score	Out-of-pocket expenditure per capita on healthcare	Domestic private health expenditure per capita	HIV/AIDS prevalence	Air transport passengers carried
Physician density	1.00	0.67	0.47	0.57	0.59	0.51	0.55	0.56	0.65	0.56	-0.20	0.34
Health worker density	0.67	1.00	0.55	0.48	0.55	0.48	0.56	0.58	0.59	0.52	-0.27	0.40
Nurse and midwife density	0.47	0.55	1.00	0.55	0.54	0.46	0.87	0.77	0.75	0.79	-0.20	0.55
Hospital beds	0.57	0.48	0.55	1.00	0.41	0.45	0.65	0.51	0.59	0.59	-0.34	0.26
COVID-19 boosters	0.59	0.55	0.54	0.41	1.00	0.27	0.47	0.43	0.49	0.45	-0.18	0.23
RCV1	0.51	0.48	0.46	0.45	0.27	1.00	0.50	0.45	0.47	0.38	-0.28	0.20
Median income or expenditure	0.55	0.56	0.87	0.65	0.47	0.50	1.00	0.85	0.82	0.82	-0.31	0.48
Cantril ladder score	0.56	0.58	0.77	0.51	0.43	0.45	0.85	1.00	0.71	0.71	-0.45	0.40
Out-of-pocket expenditure per capita on healthcare	0.65	0.59	0.75	0.59	0.49	0.47	0.82	0.71	1.00	0.89	-0.16	0.45
Domestic private health expenditure per capita	0.56	0.52	0.79	0.59	0.45	0.38	0.82	0.71	0.89	1.00	-0.06	0.59
HIV/AIDS prevalence	-0.20	-0.27	-0.20	-0.34	-0.18	-0.28	-0.31	-0.45	-0.16	-0.06	1.00	0.29
Air transport passengers carried	0.34	0.40	0.55	0.26	0.23	0.20	0.48	0.40	0.45	0.59	0.29	1.00

Supplementary Table S3. Timeline of first policy implementation in countries affected by Mpox

Countries	Time	Source
Belgium	20/05/2022	https://www.cnbc.com/2022/05/23/belgium-introduces-mandatory-monkeypox-quarantine-as-global-cases-rise.html
UK	30/05/2022	https://www.gov.uk/government/news/public-health-agencies-issue-monkeypox-guidance-to-control-transmission
Netherlands	24/05/2022	https://www.government.nl/latest/news/2022/05/24/monkeypox-cases-must-be-reported#:~:text=Health%20minister%20Ernst%20Kuipers%20designated%20the%20monkeypox%20as,cases%20immediately%20to%20prevent%20the%20virus%20from%20spreading
United Arab Emirates	01/08/2022	https://www.dha.gov.ae/uploads/062022/monkeypox guideline_EN2022639203.pdf
Canada	27/05/2022	https://www.canada.ca/en/public-health/services/diseases/mpox/health-professionals/interim-guidance-infection-prevention-control-healthcare-settings.html#a4
Austria	24/05/2022	https://www.thelocal.com/20220524/austria-makes-quarantine-announcement-for-monkeypox/
Chili	26/06/2022	https://outbreaknewstoday.com/chile-ministry-of-health-decrees-sanitary-alert-for-monkeypox-57874/
Denmark	02/12/2022	https://www.sst.dk/en/english/Expertise-and-guidance/General-public/Monkeypox
Dominica	23/05/2022	https://dominicantoday.com/dr/local/2022/05/23/public-health-vigilant-against-monkeypox/
France	20/05/2022	https://www.santepubliquefrance.fr/maladies-et-traumatismes/maladies-transmissibles-de-l-animal-a-l-homme/monkeypox
Ghana	11/06/2022	https://yen.com.gh/ghana/208383-monkeypox-in-ghana-symptoms-treatment-and-how-to-avoid-contracting-the-disease/
Greece	02/09/2022	https://greekcitytimes.com/2022/09/02/covid-19-monkeypox-buruli-ulcer-tomato-flu-and-other-outbreaks-all-you-need-to-know/

Ireland	20/05/2022	https://www.gov.ie/en/press-release/5b664-minister-for-health-announces-updates-to-irelands-monkeypox-vaccine-strategy/
Italy	26/05/2022	https://www.thelocal.it/20220526/explained-how-is-italy-dealing-with-rising-monkeypox-cases
Luxembourg	04/08/2022	https://msan.gouvernement.lu/en/actualites.gouvernement%2Ben%2Bactualites%2Btoutes_actualites%2Bcommuniques%2B2022%2B08-aout%2B04-variole-singe.html#:~:text=The%20strategy%20to%20reduce%20human-to-human%20transmission%20of%20monkeypox,diagnostic%20confirmation%20by%20PCR%3B%20Isolation%20of%20infected%20patients%3B
Nigeria	25/05/2022	https://theconversation.com/monkeypox-is-endemic-in-nigeria-but-surveillance-isnt-what-it-should-be-183711
Norway	25/07/2022	https://www.fhi.no/publ/2022/apekopper-risikovurderinger/
Paraguay	18/11/2022	https://www.plenglish.com/news/2022/11/18/paraguay-takes-measures-to-prevent-spread-of-monkeypox/
Peru	20/05/2022	https://english.news.cn/20220520/b79695dfc0aa4e2297fe8185898a2b2e/c.html
Poland	23/05/2022	https://www.thefirstnews.com/article/no-major-threat-of-monkeypox-in-poland---health-ministry-30527
Spain	05/07/2022	https://euroweeklynews.com/2022/07/05/spains-ministry-of-health-tightens-monkeypox-virus-protocol-as-cases-rise/
Sweden	22/05/2022	https://www.politifact.com/factchecks/2022/may/25/facebook-posts/sweden-isnt-planning-monkeypox-restrictions-those-/
Switzerland	27/05/2022	https://www.bag.admin.ch/bag/en/home/krankheiten/krankheiten-im-ueberblick/mpox.html
USA	28/06/2022	https://www.whitehouse.gov/briefing-room/statements-releases/2022/06/28/fact-sheet-biden-harris-administrations-monkeypox-outbreak-response/

Supplementary Table S4. Number of cases in the 39 countries severely affected by Mpox

data	Argentina	Austria	Belgium	Bolivia	Brazil	Canada	Chile	Colombia	Costa Rica	Czechia	Democratic Republic of Congo	Denmark	Dominican Republic	Ecuador	El Salvador	France	Germany	Ghana	Greece	Guadeloupe	Hungary	Iceland	Israel	Italy	Luxembourg	Mexico	Netherlands	Nigeria	Norway	Panama	Paraguay	Peru	Poland	Portugal	Spain	Sweden	Switzerland	United Kingdom	United States
15/05/2022	0	0	0	0	0	0	0	0	0	0	0	0	0	0	0	0	0	2	0	0	0	0	0	0	0	0	0	19	0	0	0	0	0	0	0	0	0	3	0
22/05/2022	0	0	3	0	0	0	0	0	0	0	10	0	0	0	0	1	3	2	0	0	0	0	1	3	0	0	2	21	0	0	0	0	0	37	40	1	1	29	0
29/05/2022	0	1	6	0	0	0	0	0	0	5	10	2	0	0	0	7	15	2	0	0	0	0	1	8	0	0	26	21	0	0	0	0	0	74	40	2	3	106	0
05/06/2022	2	1	17	0	0	77	0	0	0	6	10	2	0	0	0	51	65	8	0	0	1	0	2	20	0	1	40	32	2	0	0	0	0	143	186	5	8	225	25
12/06/2022	3	1	24	0	1	112	0	0	0	6	10	3	0	0	0	91	165	19	0	0	2	0	4	20	0	1	60	36	2	0	0	0	0	209	259	6	14	366	49
19/06/2022	3	11	62	0	7	177	1	0	0	6	100	8	0	0	0	183	338	22	2	0	6	3	6	71	1	10	95	41	4	0	0	0	1	276	497	10	31	574	113
26/06/2022	4	20	77	0	19	235	3	3	0	7	100	13	0	0	0	330	676	23	3	0	12	3	18	127	3	11	211	63	4	0	0	0	7	348	736	13	55	910	199
03/07/2022	6	37	117	0	63	287	6	5	0	8	100	20	0	0	0	498	1054	29	3	0	18	4	50	192	3	27	288	84	15	0	0	3	10	415	800	28	115	1235	396
10/07/2022	6	62	169	0	205	375	13	6	0	10	100	28	1	1	0	721	1490	34	12	0	24	6	61	255	6	30	402	101	19	1	0	18	19	473	2034	43	140	1552	699
17/07/2022	13	74	225	0	384	539	16	7	0	11	100	33	1	1	0	912	1790	36	13	0	28	7	80	292	8	42	549	118	35	1	0	55	22	515	2447	58	189	1735	1466
24/07/2022	18	91	312	0	592	681	20	12	1	14	100	51	3	3	0	1453	2268	37	20	0	33	9	105	374	14	59	712	133	46	1	0	143	40	588	3125	77	216	2137	2582
31/07/2022	20	118	393	0	978	803	55	12	3	16	100	81	3	3	0	1837	2595	51	32	0	37	9	121	426	23	59	879	157	51	1	0	269	53	633	3738	85	264	2432	4897
07/08/2022	31	154	482	3	1721	957	68	43	3	26	100	108	4	7	0	2239	2887	55	39	1	42	10	160	505	31	91	959	172	64	2	0	340	63	710	4577	111	312	2759	7510
14/08/2022	49	199	546	11	2584	1059	141	84	3	35	149	141	5	17	0	2673	3102	67	49	1	57	11	174	644	41	147	1025	220	70	2	0	653	95	770	5719	129	376	3037	10725
21/08/2022	72	217	624	37	3450	1168	207	164	3	36	149	158	6	19	0	2889	3266	78	50	1	62	12	194	689	45	252	1087	241	76	7	0	937	104	810	5792	139	392	3195	14049
28/08/2022	133	258	671	55	4472	1228	270	273	3	46	174	171	7	44	0	3421	3405	85	54	1	67	12	215	740	50	386	1136	277	79	8	1	1300	128	846	6459	156	431	3340	16965
04/09/2022	170	273	706	86	5197	1289	381	582	3	53	174	177	7	53	1	3646	3493	88	58	1	70	12	239	773	53	504	1172	318	82	11	1	1546	141	871	6645	163	476	3413	19351
11/09/2022	221	286	726	103	5971	1321	486	938	3	62	174	181	21	59	1	3785	3530	92	66	1	71	12	245	805	54	788	1195	349	85	12	1	1808	156	898	6892	174	484	3484	21504
18/09/2022	265	301	744	129	6807	1363	728	1260	4	65	190	183	31	68	2	3898	3556	95	69	1	75	12	246	828	55	1050	1209	400	89	12	1	2054	167	908	6947	179	498	3552	22957
25/09/2022	326	307	757	164	7418	1389	842	1653	4	66	200	183	31	93	4	3943	3597	100	72	1	77	12	250	842	55	1367	1219	456	91	13	1	2311	173	917	7083	186	503	3585	24402
02/10/2022	396	313	770	195	7869	1396	880	2042	4	67	202	185	31	120	7	3999	3625	104	80	1	77	14	250	850	55	1627	1219	481	92	16	2	2521	188	926	7188	195	513	3635	25434
09/10/2022	479	317	775	215	8340	1410	977	2453	6	70	228	187	52	142	9	4043	3645	104	82	1	78	16	254	854	55	1968	1221	530	92	16	2	25	195	930	72	198	517	3654	26393

																																87			09				
16/10/2022	524	320	779	231	8652	1410	1064	2730	7	70	235	189	52	185	10	4064	3651	105	82	1	78	16	254	881	55	2147	1223	552	92	16	2	2785	196	940	7239	206	521	3654	27128
23/10/2022	620	323	785	239	8978	1435	1116	3110	8	70	250	191	52	212	11	4084	3656	105	82	1	79	16	257	890	55	2468	1230	583	92	16	2	2942	201	944	7277	207	544	3686	27689
30/10/2022	675	323	785	241	9183	1435	1149	3298	8	70	251	191	52	243	13	4094	3662	107	84	1	80	16	260	894	55	2654	1235	604	93	16	3	3048	206	944	7317	209	546	3698	28049
06/11/2022	746	325	785	242	9312	1444	1175	3523	8	70	252	191	52	243	17	4097	3668	107	84	1	80	16	262	898	55	2901	1237	624	93	18	5	3204	210	944	7336	212	546	3701	28423
13/11/2022	746	325	785	247	9606	1444	1226	3630	16	70	258	191	52	311	17	4102	3670	107	85	1	80	16	262	915	56	3007	1240	638	93	21	7	3299	212	948	7377	212	546	3703	28683
20/11/2022	865	326	786	251	9844	1449	1259	3719	23	70	262	191	52	346	21	4104	3671	107	85	1	80	16	262	917	57	3145	1247	659	93	34	16	3359	212	948	7392	216	546	3710	28935
27/11/2022	894	326	789	252	9935	1449	1278	3803	24	70	272	191	52	346	21	4107	3671	110	85	1	80	16	262	918	57	3292	1248	680	93	48	17	3444	213	948	7405	220	546	3720	29001
04/12/2022	944	327	789	259	10100	1456	1312	3861	24	71	275	191	52	346	21	4110	3671	110	85	1	80	16	262	921	57	3362	1251	720	93	56	26	3508	214	948	7408	229	550	3725	29432
11/12/2022	972	327	790	260	10235	1459	1340	3880	37	71	275	191	52	346	30	4110	3673	116	85	1	80	16	262	927	57	3455	1251	744	93	56	34	3566	214	948	7412	236	551	3730	29513
18/12/2022	987	327	790	261	10293	1459	1361	3908	37	71	277	192	52	346	40	4110	3675	116	85	1	80	16	262	932	57	3509	1256	753	93	66	38	3587	214	948	7416	242	551	3730	29513
25/12/2022	987	327	790	261	10448	1460	1372	3971	37	71	279	192	52	346	52	4110	3676	121	85	1	80	16	262	936	57	3509	1256	756	93	78	39	3629	214	948	7496	250	551	3730	29554
01/01/2023	1018	327	790	261	10511	1460	1377	4021	70	71	279	192	52	441	69	4114	3676	121	85	1	80	16	262	938	57	3637	1256	763	93	78	48	3643	214	948	7496	250	551	3730	29603
08/01/2023	1025	327	790	262	10589	1460	1387	4035	77	71	316	192	52	441	69	4114	3677	121	85	1	80	16	262	951	57	3637	1259	766	94	82	56	3683	214	950	7500	255	551	3730	29724
15/01/2023	1037	327	790	263	10625	1460	1398	4049	77	71	333	192	52	460	69	4114	3684	121	85	1	80	16	262	951	57	3696	1260	776	94	82	63	3698	214	950	7505	255	551	3730	29787
22/01/2023	1052	327	790	263	10690	1460	1406	4062	126	71	355	192	52	460	69	4114	3689	123	86	1	80	16	262	954	57	3696	1260	787	94	99	68	3716	215	950	7514	257	551	3735	29849
29/01/2023	1064	327	790	263	10730	1460	1408	4066	140	71	377	194	52	478	88	4128	3692	123	86	1	80	16	262	954	57	3768	1260	790	94	110	82	3723	215	950	7518	257	551	3735	29891
05/02/2023	1075	327	793	264	10758	1460	1417	4072	140	71	387	196	52	483	88	4128	3692	123	86	1	80	16	262	955	57	3768	1260	797	95	118	82	3727	215	951	7528	260	551	3735	29933
12/02/2023	1089	327	793	264	10808	1460	1423	4074	140	71	387	196	52	497	88	4128	3692	123	86	1	80	16	262	957	57	3828	1260	801	95	129	92	3737	215	951	7533	260	551	3735	29974
19/02/2023	1095	327	793	264	10808	1460	1427	4080	178	71	420	196	52	502	88	4128	3692	123	86	1	80	16	262	957	57	3828	1261	806	95	139	98	3752	215	951	7538	260	551	3735	29987

Supplementary Table S5. Statistical tests and initial variable selection of socioeconomic, environmental, and meteorological factors

Items	Lasso regression coefficient	Skewness	Kurtosis	Kolmogorov–Smirnov test		Shapiro–Wilk test	
				Statistic *D*	*p*	Statistic *W*	*p*
Cumulative confirmed cases of Mpox	-	4.624	24.240	0.338	0.000**	0.430	0.000**
Cumulative prevalence of Mpox (millions)	-	1.298	1.993	0.154	0.020*	0.887	0.001**
Prevalence of asthma	0.28	0.824	0.548	0.166	0.008**	0.944	0.051
Tumour mortality	0.088	0.401	-0.244	0.067	0.928	0.973	0.462
Incidence of tumours	-0.328	3.290	15.689	0.178	0.003**	0.699	0.000**
Under a mortality rate of 5	0.084	3.400	12.527	0.306	0.000**	0.534	0.000**
COVID-19 mortality	-0.039	0.708	1.825	0.114	0.232	0.951	0.087
COVID-19 confirmation	0.247	0.274	-1.147	0.108	0.298	0.943	0.049*
COVID-19 boosters	-0.313*	0.552	1.758	0.091	0.576	0.964	0.233
Mortality due to household air pollution from solid fuels	0.015	3.593	14.277	0.373	0.000**	0.467	0.000**
Air pollution mortality	0.083	1.351	1.836	0.123	0.146	0.883	0.001**
Meningitis mortality	-0.013	3.730	13.676	0.414	0.000**	0.383	0.000**
Overweight mortality	0.063	0.720	-0.681	0.152	0.024*	0.905	0.003**
Opioid use disorder mortality	-0.036	4.337	22.335	0.303	0.000**	0.508	0.000**
Poisoning mortality	0.14	3.201	11.159	0.357	0.000**	0.558	0.000**

Items	Lasso regression coefficient	Skewness	Kurtosis	Kolmogorov–Smirnov test		Shapiro–Wilk test	
				Statistic *D*	*p*	Statistic *W*	*p*
Unsafe sanitation mortality	0.07	3.927	15.794	0.433	0.000**	0.366	0.000**
Venomous animal contact mortality	0.088	3.012	8.306	0.345	0.000**	0.483	0.000**
Lead exposure mortality	-0.182	1.759	2.822	0.181	0.002**	0.791	0.000**
Ambient particulate matter pollution mortality	0.036	1.564	3.316	0.128	0.113	0.873	0.000**
Air pollution mortality	-0.052	2.450	5.935	0.265	0.000**	0.673	0.000**
Ambient ozone pollution mortality	-0.071	1.455	3.227	0.136	0.068	0.888	0.001**
Alcohol use disorder mortality	-0.146	1.479	2.051	0.150	0.028*	0.840	0.000**
Mental and substance use disorders mortality	-0.06	1.830	2.789	0.220	0.000**	0.746	0.000**
Road injuries mortality	-0.261	1.479	2.826	0.196	0.001**	0.853	0.000**
Substance use disorder mortality	0.095	2.121	5.376	0.196	0.001**	0.778	0.000**
Proportion of alcohol in the diet	-0.091	1.056	1.411	0.127	0.119	0.924	0.012*
Drowning mortality	0.069	0.660	-0.684	0.164	0.010*	0.899	0.002**
HAQ Index	0.161	-0.734	-0.558	0.218	0.000**	0.881	0.001**
Interpersonal violence mortality	-0.105	3.433	14.476	0.294	0.000**	0.579	0.000**
Hospital beds	0.199*	0.846	-0.223	0.151	0.025*	0.912	0.005**
HepB3	0.095	-0.865	-0.124	0.188	0.001**	0.904	0.003**

Items	Lasso regression coefficient	Skewness	Kurtosis	Kolmogorov–Smirnov test		Shapiro–Wilk test	
				Statistic *D*	*p*	Statistic *W*	*p*
Protein-energy malnutrition mortality	-0.185	2.363	6.436	0.247	0.000**	0.695	0.000**
Maternal mortality	0.125	4.090	18.611	0.337	0.000**	0.452	0.000**
Mean BMI (male)	0.076	-1.858	4.259	0.179	0.003**	0.818	0.000**
Mean BMI (female)	0.018	-0.426	-0.396	0.097	0.473	0.976	0.556
Median age	-0.103	-0.595	-0.689	0.168	0.007**	0.925	0.013*
Pol3	-0.073	-0.996	-0.173	0.243	0.000**	0.844	0.000**
RCV1	0.504*	-1.210	0.806	0.185	0.002**	0.867	0.000**
MCV1	-0.043	-1.359	1.237	0.192	0.001**	0.842	0.000**
UHC Service Coverage Index	-0.025	-1.751	2.694	0.195	0.001**	0.793	0.000**
Prevalence of zinc deficiency	-0.061	2.757	9.750	0.243	0.000**	0.702	0.000**
Alzheimer's disease and other dementia mortality	-0.005	0.499	-0.883	0.188	0.001**	0.925	0.012*
Diabetes mortality	-0.122	1.314	0.965	0.248	0.000**	0.823	0.000**
Diarrheal disease mortality	-0.149	3.962	16.592	0.406	0.000**	0.405	0.000**
Incidence of acute hepatitis B	-0.163	3.226	10.646	0.309	0.000**	0.533	0.000**
Acute hepatitis mortality	-0.046	2.973	8.108	0.353	0.000**	0.474	0.000**
HIV/AIDS prevalence	-0.499**	3.521	12.584	0.318	0.000**	0.489	0.000**

Items	Lasso regression coefficient	Skewness	Kurtosis	Kolmogorov–Smirnov test		Shapiro–Wilk test	
				Statistic *D*	*p*	Statistic *W*	*p*
CVD mortality	-0.165	1.164	0.532	0.253	0.000**	0.851	0.000**
Prevalence of anaemia among pregnant women	0.048	2.420	5.909	0.274	0.000**	0.696	0.000**
Chronic respiratory disease mortality	0.019	1.848	5.642	0.116	0.205	0.862	0.000**
Cardiovascular disease mortality	0.044	1.280	0.838	0.169	0.007**	0.847	0.000**
Prevalence of schizophrenia	0.25	1.422	5.123	0.225	0.000**	0.827	0.000**
Prevalence of autism spectrum disorders	0.074	0.400	-1.168	0.215	0.000**	0.882	0.001**
Prevalence of anxiety disorders	0.099	0.199	-0.600	0.067	0.927	0.979	0.659
Prevalence of bipolar disorder	0.128	-0.663	-0.350	0.141	0.047*	0.916	0.006**
Prevalence of depressive disorders	0.051	0.073	0.225	0.116	0.203	0.967	0.310
Prevalence of Down syndrome	0.071	0.002	-1.352	0.226	0.000**	0.903	0.003**
Prevalence of idiopathic developmental intellectual disability	-0.026	2.778	9.437	0.248	0.000**	0.688	0.000**
Stroke mortality	-0.104	1.874	3.601	0.232	0.000**	0.784	0.000**
Tetanus mortality	0.031	4.088	17.991	0.396	0.000**	0.382	0.000**
Tuberculosis mortality	-0.037	3.946	16.481	0.395	0.000**	0.387	0.000**
Total dependency ratio	0.022	2.454	8.071	0.258	0.000**	0.752	0.000**
Old age dependency ratio	-0.055	-0.162	-1.511	0.183	0.002**	0.906	0.003**

Items	Lasso regression coefficient	Skewness	Kurtosis	Kolmogorov–Smirnov test		Shapiro–Wilk test	
				Statistic *D*	*p*	Statistic *W*	*p*
Meat supply per capita	-0.325	0.986	0.182	0.181	0.002**	0.889	0.001**
DALYs	0.026	1.514	4.574	0.137	0.061	0.896	0.002**
DALYs age: 70+ yrs	0.027	0.236	-1.377	0.171	0.006**	0.914	0.006**
DALYs age: 50–69 yrs	0.113	1.263	2.065	0.159	0.015*	0.896	0.002**
DALYs age: 15–49 yrs	0.072	1.375	1.367	0.178	0.003**	0.831	0.000**
DALYs age: 5–14 yrs	-0.265	1.498	2.403	0.224	0.000**	0.803	0.000**
DALYs under 5 yrs	-0.179	2.611	7.413	0.263	0.000**	0.641	0.000**
Meat supply per capita	0.03	-0.643	0.659	0.139	0.056	0.952	0.093
Armed force personnel	0.029	2.837	9.840	0.209	0.000**	0.694	0.000**
Automated teller machines	0.179	0.904	0.498	0.137	0.062	0.935	0.027*
Average childbearing age	-0.181	-0.454	-0.983	0.150	0.026*	0.933	0.022*
Average harmonised learning outcome score	0.244	-1.190	0.901	0.256	0.000**	0.839	0.000**
Height	0.236	-0.446	-0.454	0.115	0.214	0.970	0.382
Births per woman	0.189	2.981	9.582	0.268	0.000**	0.620	0.000**
Mean height (female)	-0.184	-0.544	-0.674	0.190	0.001**	0.932	0.021*
Child dependency ratio	-0.058	2.104	4.908	0.221	0.000**	0.763	0.000**

Items	Lasso regression coefficient	Skewness	Kurtosis	Kolmogorov–Smirnov test		Shapiro–Wilk test	
				Statistic *D*	*p*	Statistic *W*	*p*
Democracy satisfaction index	-0.292	0.804	-0.016	0.153	0.022*	0.929	0.016*
Volunteerism index	0.093	-0.987	-0.292	0.186	0.002**	0.850	0.000**
Administrative index	-0.22	-2.426	5.900	0.390	0.000**	0.572	0.000**
Birth rate	0.241	2.171	5.273	0.217	0.000**	0.756	0.000**
Median income or expenditure	-0.273*	0.260	-1.392	0.154	0.021*	0.915	0.006**
Proportion of fat in the diet	-0.077	-0.682	-0.535	0.179	0.003**	0.923	0.011*
Proportion of protein in the diet	-0.115	-1.072	1.301	0.181	0.002**	0.911	0.005**
Proportion of vegetable oils in the diet	-0.213	0.129	-0.674	0.070	0.896	0.971	0.407
Proportion of oil crops in the diet	0.13	1.666	2.687	0.223	0.000**	0.825	0.000**
Proportion of fish and seafood in the diet	0.168	0.906	0.608	0.116	0.209	0.924	0.011*
Proportion of sugar and sweeteners in the diet	-0.139	-0.538	0.688	0.087	0.642	0.966	0.287
Proportion of starchy roots in the diet	0.03	3.261	10.751	0.376	0.000**	0.516	0.000**
Proportion of pork in the diet	0.138	0.184	-1.109	0.134	0.073	0.944	0.050
Proportion of poultry in the diet	0.01	0.517	-0.196	0.115	0.216	0.960	0.179
Proportion of beef in the diet	0.079	2.045	6.782	0.140	0.051	0.837	0.000**
Proportion of eggs in the diet	0.183	-0.545	0.607	0.114	0.227	0.966	0.287

Items	Lasso regression coefficient	Skewness	Kurtosis	Kolmogorov–Smirnov test		Shapiro–Wilk test	
				Statistic *D*	*p*	Statistic *W*	*p*
Proportion of milk in the diet	-0.022	-0.242	-0.467	0.086	0.659	0.976	0.557
Proportion of nuts in the diet	0.228	0.540	-0.938	0.163	0.011*	0.896	0.002**
Proportion of fruit in the diet	-0.022	3.090	11.033	0.265	0.000**	0.651	0.000**
Proportion of vegetables in the diet	0.116	-0.361	-0.303	0.120	0.171	0.973	0.459
Proportion of pulses in the diet	-0.03	1.012	0.229	0.184	0.002**	0.886	0.001**
Proportion of maize in the diet	0.018	2.198	4.587	0.227	0.000**	0.707	0.000**
Proportion of rice in the diet	-0.027	2.008	4.193	0.285	0.000**	0.719	0.000**
Proportion of wheat in the diet	-0.108	-0.237	-1.207	0.129	0.099	0.937	0.030*
Duration of compulsory education	0.047	-0.152	-0.189	0.124	0.136	0.962	0.205
Election democracy index	-0.11	-1.008	0.027	0.185	0.002**	0.869	0.000**
Parity index	0.011	-0.575	-1.310	0.231	0.000**	0.847	0.000**
Female employment to population ratio	-0.029	-0.041	-0.915	0.116	0.210	0.968	0.336
Percentage of girls in primary education	-0.128	-0.594	0.323	0.113	0.234	0.962	0.214
Gender inequality index	-0.021	0.692	-0.695	0.215	0.000**	0.880	0.001**
Government transparency	0.112	-0.154	-0.892	0.121	0.157	0.948	0.068
Gross enrolment rate of preschool education	-0.139	-0.926	0.718	0.130	0.097	0.929	0.017*

Items	Lasso regression coefficient	Skewness	Kurtosis	Kolmogorov–Smirnov test		Shapiro–Wilk test	
				Statistic *D*	*p*	Statistic *W*	*p*
Gross enrolment rate of primary education	0.154	1.166	2.890	0.115	0.217	0.929	0.017*
Gross enrolment rate of secondary education	0.147	-0.069	0.870	0.140	0.052	0.970	0.371
Gross enrolment rate of tertiary education	0.002	-0.165	-0.290	0.084	0.691	0.980	0.687
Cantril ladder score	-0.305*	-1.288	3.116	0.100	0.419	0.914	0.006**
Civil liberties index	-0.09	-1.509	1.475	0.237	0.000**	0.799	0.000**
Number of inbound visitors	0.009	2.582	7.422	0.275	0.000**	0.650	0.000**
Air transport passengers	0.319*	1.265	1.322	0.200	0.000**	0.857	0.000**
Top 10% income quantile	-0.046	0.429	-0.577	0.108	0.299	0.957	0.139
Bottom 10% income quantile	0.067	0.330	-1.437	0.165	0.009**	0.893	0.001**
Natural growth rate	0.048	0.595	1.077	0.126	0.125	0.958	0.150
Bank Coverage	-0.03	1.545	2.876	0.173	0.005**	0.875	0.000**
Proportion of refugees	-0.174	3.827	14.602	0.424	0.000**	0.370	0.000**
Nurse and midwife density	0.231*	0.495	-0.882	0.157	0.017*	0.918	0.008**
Out-of-pocket expenditure per capita	-0.454*	1.224	3.642	0.110	0.275	0.916	0.007**
Primary energy consumption per capita	-0.045	1.290	1.680	0.173	0.005**	0.888	0.001**
Health worker density	0.442*	0.424	-0.032	0.142	0.046*	0.946	0.059

Items	Lasso regression coefficient	Skewness	Kurtosis	Kolmogorov–Smirnov test		Shapiro–Wilk test	
				Statistic *D*	*p*	Statistic *W*	*p*
Physician density	0.282*	0.028	-0.514	0.068	0.918	0.980	0.686
Population density	0.034	2.106	5.227	0.206	0.000**	0.767	0.000**
Population in the largest city	0.042	1.144	1.944	0.148	0.032*	0.920	0.009**
Domestic private health expenditure per capita	-0.61*	3.044	10.391	0.240	0.000**	0.645	0.000**
Researcher density	0.04	0.083	-0.942	0.154	0.020*	0.920	0.009**
Urban population	0.034	-0.533	-0.367	0.138	0.059	0.962	0.207
Outbound mobility ratio	0.028	6.163	38.283	0.431	0.000**	0.211	0.000**
Corruption perception index	0.16	-0.004	-1.520	0.139	0.056	0.913	0.005**
Time required to start a business	0.149	1.584	2.153	0.182	0.002**	0.823	0.000**
Female unemployment rate	0.176	2.340	6.003	0.261	0.000**	0.721	0.000**
Total unemployment rate	0.003	1.178	0.687	0.176	0.004**	0.876	0.000**
Total water withdrawal per capita	0.013	1.941	5.335	0.181	0.002**	0.837	0.000**
Aquaculture production	0.138	2.876	8.544	0.290	0.000**	0.583	0.000**
Grain yield	0.178	-0.225	-0.495	0.063	0.963	0.981	0.757
Average number of machines	0.188	1.468	1.314	0.197	0.001**	0.785	0.000**
Account ownership	-0.063	-0.502	-1.323	0.226	0.000**	0.854	0.000**

Items	Lasso regression coefficient	Skewness	Kurtosis	Kolmogorov–Smirnov test		Shapiro–Wilk test	
				Statistic *D*	*p*	Statistic *W*	*p*
OEC ECI Ranking	0.247	0.853	-0.248	0.184	0.002**	0.899	0.002**
Gini coefficient	-0.046	0.420	-0.921	0.174	0.004**	0.946	0.058
Net FDI inflows	0.03	3.615	14.799	0.407	0.000**	0.445	0.000**
Net FDI outflows	0.059	4.342	19.754	0.457	0.000**	0.369	0.000**
GDP per capita	-0.239	1.077	1.572	0.105	0.339	0.921	0.010**
Human development index	-0.193	-1.145	0.767	0.163	0.010*	0.873	0.000**
Military expenditure	0.206	2.168	6.913	0.175	0.004**	0.821	0.000**
Military expenditure per capita	0.139	2.359	6.411	0.211	0.000**	0.727	0.000**
Research and development expenditure	0.038	0.918	0.467	0.144	0.039*	0.901	0.002**
Government expenditure on education	0.059	0.389	-0.224	0.103	0.372	0.973	0.449
Ambient PM2.5-associated mortality	0.111	1.494	2.160	0.186	0.002**	0.855	0.000**
Fertiliser use per capita	-0.202	1.907	4.810	0.188	0.001**	0.833	0.000**
Annual CO_2 emissions per capita	0.032	1.082	0.986	0.130	0.097	0.908	0.004**
Annual CO_2 emissions per unit energy	-0.17	-0.350	-0.001	0.100	0.421	0.968	0.333
Pesticide use per hectare of arable land	-0.032	1.599	3.542	0.137	0.061	0.870	0.000**
SO_2 emissions per capita	0.252	1.324	0.778	0.264	0.000**	0.788	0.000**

Items	Lasso regression coefficient	Skewness	Kurtosis	Kolmogorov–Smirnov test		Shapiro–Wilk test	
				Statistic *D*	*p*	Statistic *W*	*p*
Above-ground biomass in forest	-0.094	-0.054	-0.808	0.112	0.254	0.976	0.560
Average proportion of mountain KBAs	-0.046	-0.262	-0.885	0.128	0.109	0.955	0.119
Average proportion of freshwater KBAs	0.209	-0.222	-1.133	0.121	0.163	0.942	0.044*
Forest cover	0.069	0.134	-0.389	0.114	0.228	0.968	0.332
Ozone concentration	-0.117	0.138	-0.609	0.079	0.775	0.973	0.454
Total including LUCF per capita	-0.07	1.090	1.511	0.129	0.098	0.924	0.012*
Proportion of forest area within legally established protected areas	-0.035	0.949	0.880	0.143	0.042*	0.929	0.016*

* $p<0.05$ ** $p<0.01$

Table S1: Statistical test of monkeypox cases and socioeconomic environmental factors. When the sample size n for analysing socioeconomic and environmental factors exceeded 50, the Kolmogorov–Smirnov (K-S) test was used to verify normality [4]. Most of the factors did not comply with the K-S test, but meeting the strict requirements for the normality test was challenging. The absolute value of the kurtosis of almost all factors was < 10, and the absolute value of skewness was < 3, indicating that although the data are not absolutely normal, the distribution of the data can be accepted as a normal distribution [5,6]. COVID-19, Coronavirus Disease 2019; HAQ Index, Healthcare Access and Quality Index; HepB3, Hepatitis B; BMI, Body Mass Index; UHC, Universal health coverage; HIV/AIDS, Human immunodeficiency virus/acquired immune deficiency syndrome; CVD, Cardiovascular diseases; DALYs, Disability Adjusted Life Years; OEC ECI, The Observatory of Economic Complexity Economic Complexity Index; FDI, Foreign direct investment; GDP, Gross Domestic Product; PM2.5, Particulate matter 2.5; KBAs, Key Biodiversity Areas; LUCF, Land Use Change and Forest

Supplementary Table S6. Over-dispersion O test of three factors after the second round of variable selection

Sample size	Mean	Variance	O-value	p-value
39	41.672	1210.738	122.284	0.000

When the data had a large difference between the mean and variance and could pass the O-test with $p < 0.05$, negative binomial regression was more suitable as the analysis method than Poisson regression.

Supplementary Table S7. Collinearity test of the 12 factors after the first round of variable selection

Remaining factors after the first round of variable selection	Collinearity diagnostics	
	VIF	Tolerance
Physician density	3.652	0.274
Health worker density	2.253	0.444
Nurse and midwife density	6.730	0.149
Hospital beds	1.790	0.559
COVID-19 boosters	2.381	0.420
RCV1	1.826	0.548
Median income or expenditure	6.941	0.144
Cantril ladder score	4.132	0.242
Out-of-pocket expenditure per capita on healthcare	8.407	0.119
Domestic private health expenditure per capita	8.237	0.121
Prevalence, HIV/AIDS; Sex, Both; Age, All Ages (Number)	2.727	0.367
Air Travel log10	2.356	0.424

Tolerance $= \frac{1}{VIF}$. It is generally considered that if it is > 0.1, the collinearity of this factor is excluded [7]. VIF, Variance Inflation Factor.

Supplementary Figures

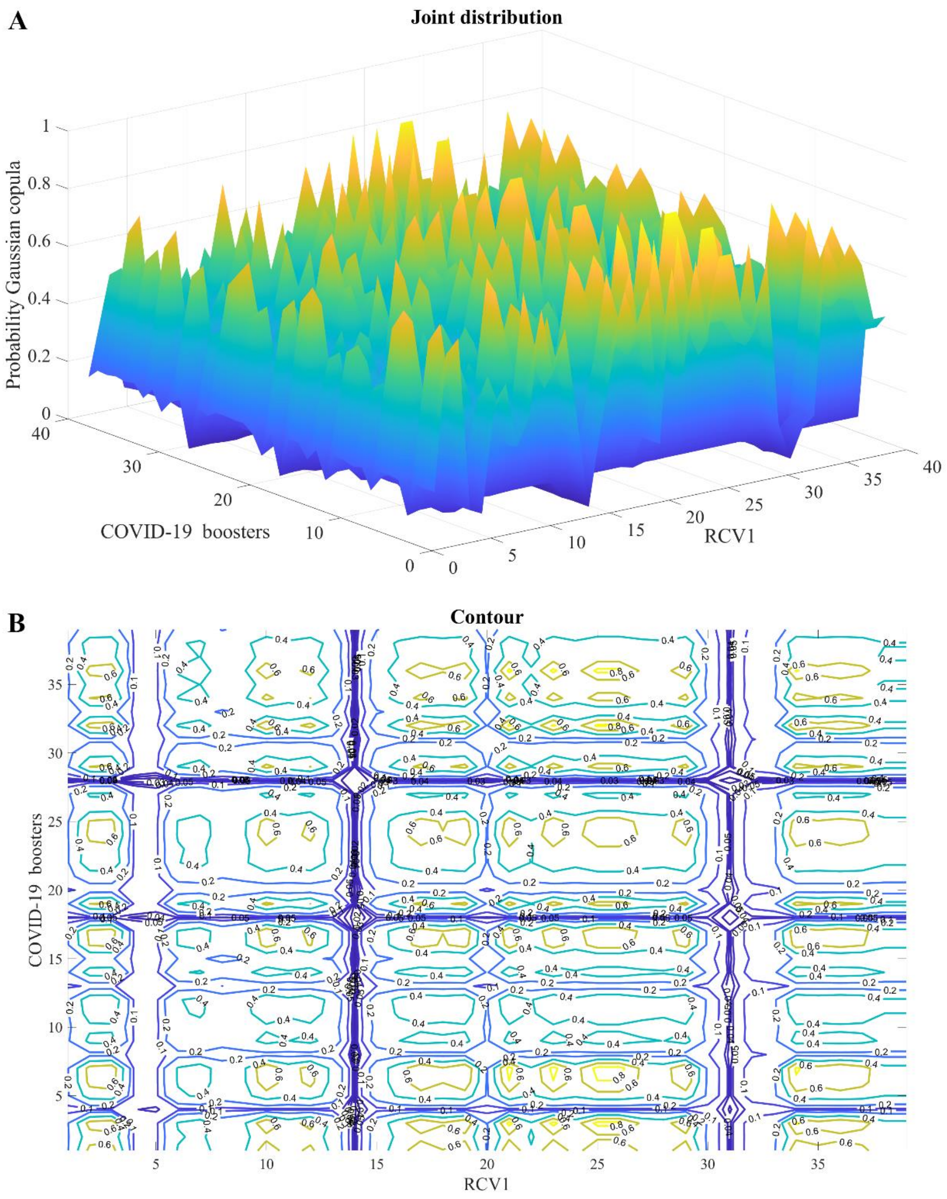

Supplementary Figure S1. (A) Gaussian-copula function joint distribution and (B) contours. A group of factors with the highest correlation coefficient of 0.85 was selected.

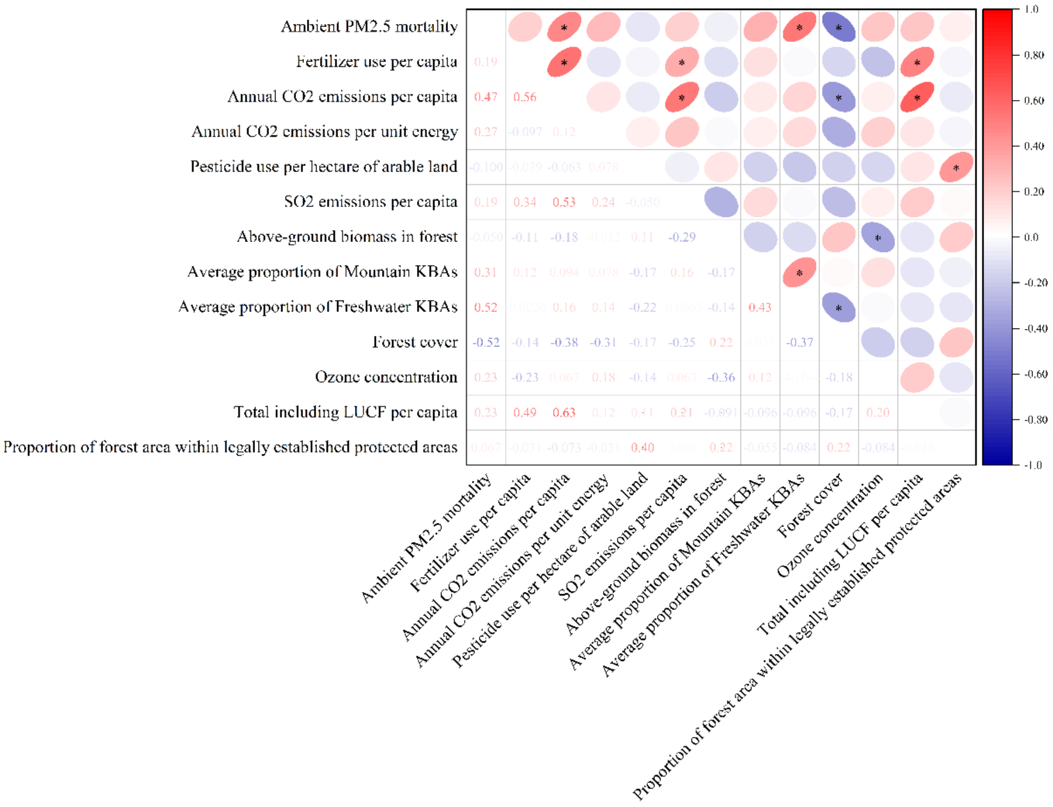

Supplementary Figure S2. Correlation coefficients of environmental factors

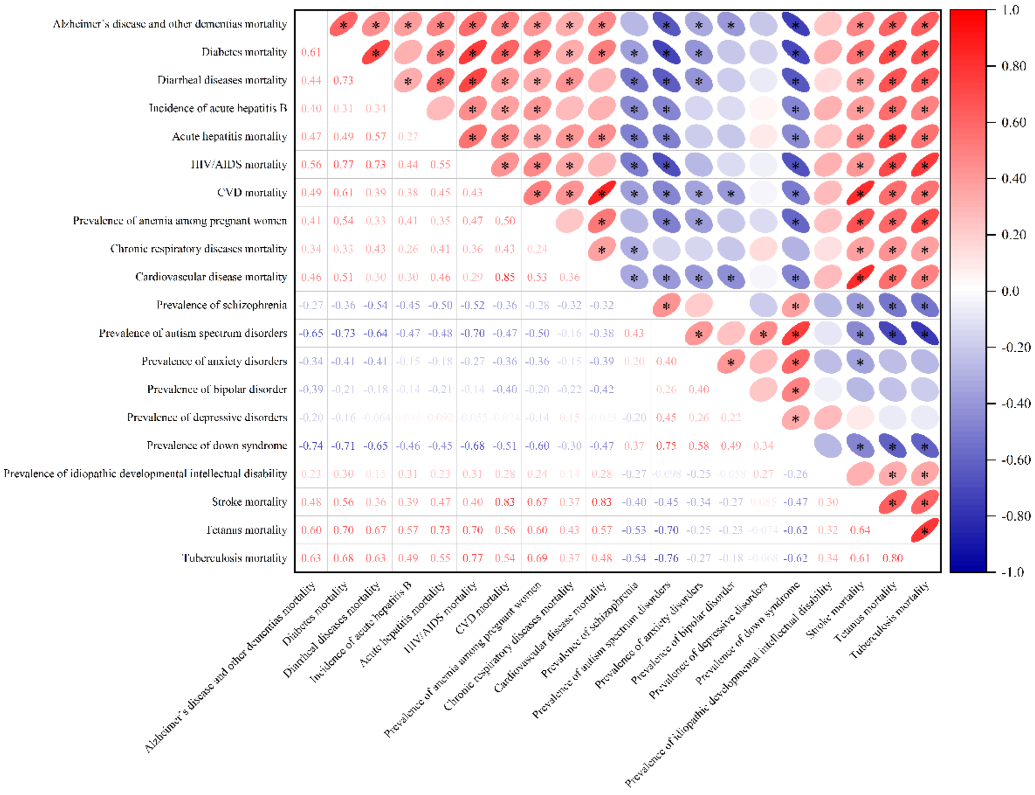

Supplementary Figure S3 Correlation coefficients of disease-related factors

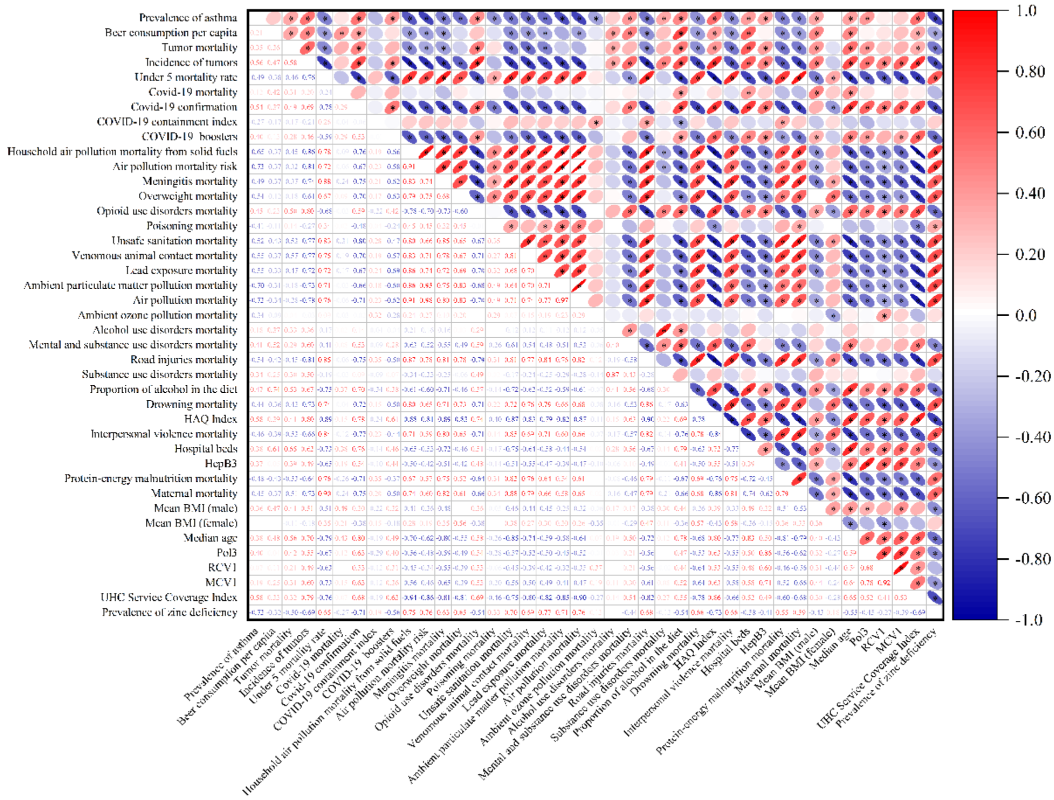

Supplementary Figure S4 Correlation coefficients of health-related factors

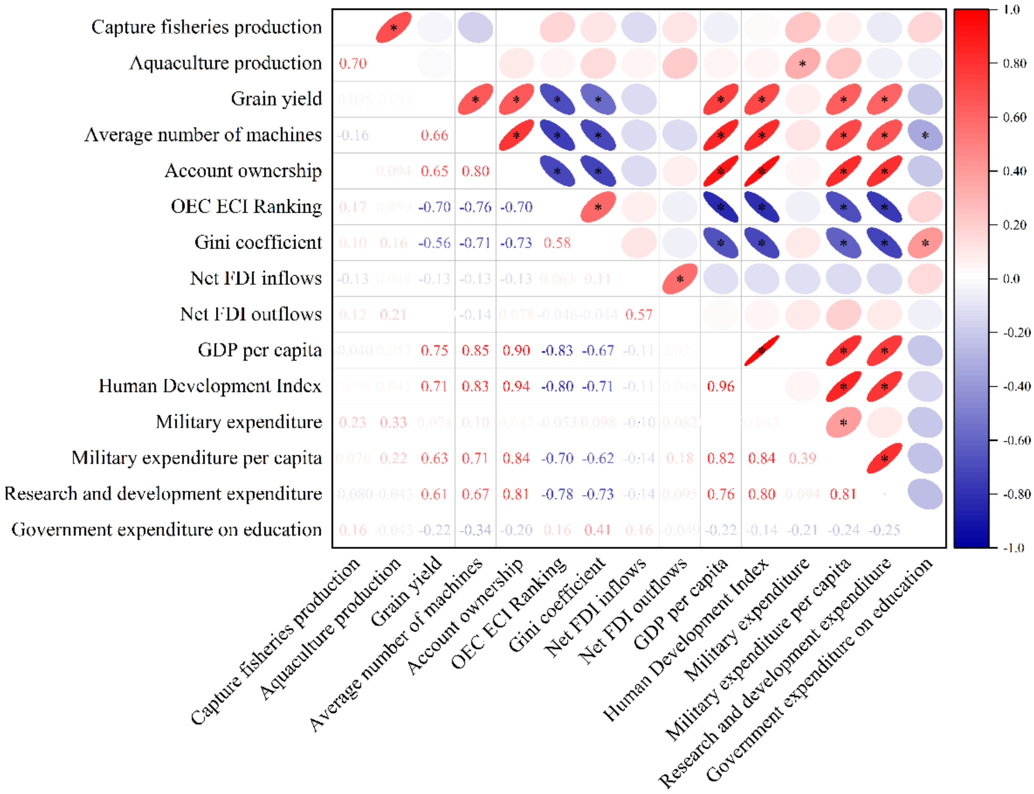

Supplementary Figure S5 Correlation coefficients of economic factors

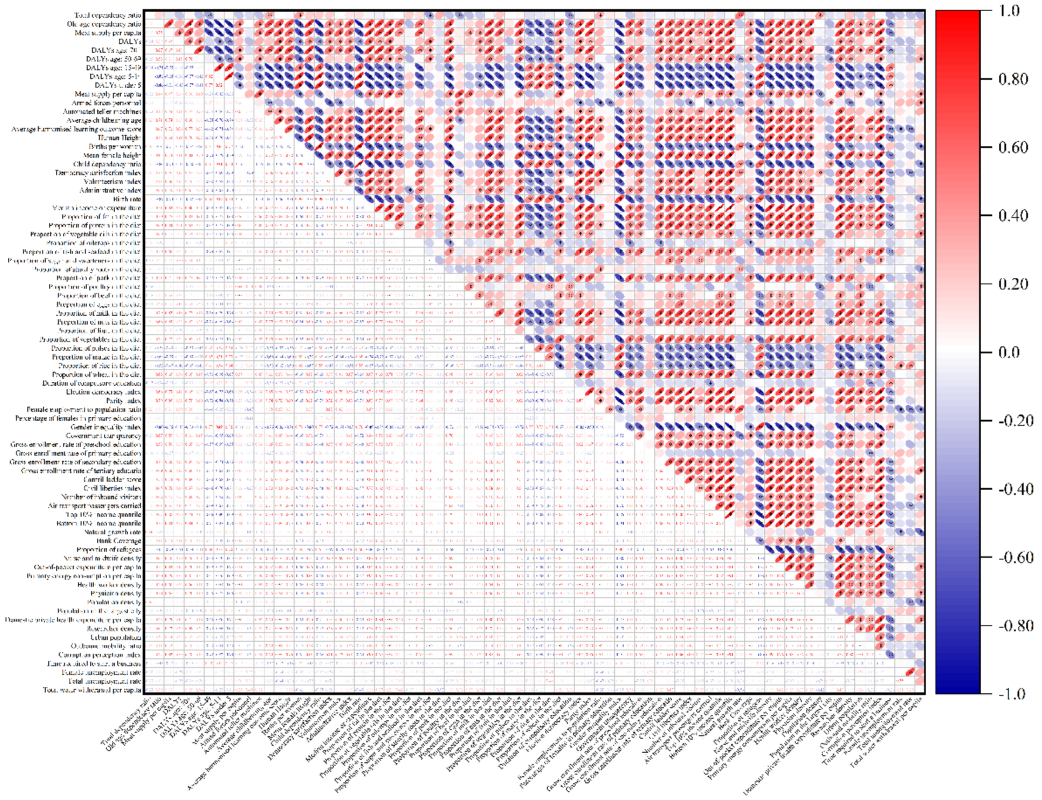

Supplementary Figure S6 Correlation coefficients of livelihood factors

References

[1] Drezner Z, Turel O, Zerom D. A modified Kolmogorov–Smirnov test for normality. Commun Stat Simul 2010;39:693–704. https://doi.org/10.1080/03610911003615816.
[2] Lilliefors HW. On the Kolmogorov-Smirnov test for normality with mean and variance unknown. J Am Stat Assoc 1967;62:399–402. https://doi.org/10.1080/01621459.1967.10482916.
[3] Kline RB. Principles and practice of structural equation modeling. Guilford publications, 2015.
[4] Drezner Z, Turel O, Zerom D. A Modified Kolmogorov-Smirnov test for normality. Commun Stat B: Simul Comput 2010;39:693–704. https://doi.org/10.1080/03610911003615816.
[5] Lilliefors HW. On the Kolmogorov-Smirnov test for normality with mean and variance unknown. J Am Stat Assoc 1967;62:399–402. https://doi.org/10.1080/01621459.1967.10482916.
[6] Kline RB. Principles and practice of structural equation modelling. J Am Stat Assoc 2011;101.
[7] Craney TA, Surles JG. Model-dependent variance inflation factor cutoff values. Qual Eng 2002;14:391–403. https://doi.org/10.1081/QEN-120001878.
